# Theoretical and experimental investigation of hydration behavior of choline salicylate ionic liquid in the presence of *L*- glycine

**DOI:** 10.1038/s41598-025-95345-8

**Published:** 2025-04-22

**Authors:** Negin Ammari, Hemayat Shekaari, Behrang Golmohammadi, Fariba Ghaffari, Mohammed Taghi Zafarani-Moattar

**Affiliations:** Department of Physical Chemistry, University of Tabriz, Tabriz, Iran

**Keywords:** Ionic liquid, Choline salicylate, *L*-glycine, Apparent molar properties, Thermodynamic and transport properties, Molar conductivity, Density functional theory, Thermodynamics

## Abstract

Choline salicylate [Ch][Sal] an active pharmaceutical ingredient ionic liquid (API-IL) aqueous solutions in the presence of *L*-glycine that is a simple simulated biological media has been studied. The thermodynamic and transport properties including density, speed of sound, viscosity and electrical conductance have been studied under atmospheric pressure and a temperature range of (288.15 to 318.15) K. The key thermophysical properties, including apparent molar volume (*V*_*φ*_), apparent molar isentropic compressibility (*κ*_*φ*_), viscosity *B*-coefficient, ion association constant (*K*_*a*_), and limiting molar conductivity (*Λ*_*0*_) were derived from these data. The transfer properties (*Δ*_*tr*_*V*_*φ*_^*0*^, *Δ*_*tr*_*κ*_*φ*_^*0*^, and *ΔB*_*tr*_) demonstrate the dominance hydrophilic-hydrophilic interactions between the IL and *L*-glycine in the studied systems that increased with *L*-glycine concentration. The COSMO results revealed that differences in molecular size, stability, and hydration behavior of [Ch][Sal] and *L*-glycine, with [Ch][Sal] exhibiting stronger hydration due to its larger size and hydrogen bonding capacity. These findings provide insights into solute-solvent interactions and potential synergistic effects of [Ch][Sal] and *L*-glycine as a simple simulated biological media contributing to the optimization of pharmaceutical formulations and related activities.

**Supplementary Information:**

The online version contains supplementary material available at 10.1038/s41598-025-95345-8.

## Introduction

Choline salicylate, a combination of choline, a B_4_-like vitamin, and salicylate, a nonsteroidal anti-inflammatory drug (NSAID), has garnered significant attention for its diverse applications in pain management and oral care^1,2^. Its analgesic and anti-inflammatory properties make it a valuable ingredient in various oral care products, while its topical formulations provide relief from conditions such as arthritis and muscle pain^3,4^. However, to ensure formulation stability and optimize therapeutic outcomes, a comprehensive understanding of its thermodynamic and transport properties in aqueous solutions is essential.

In recent years, researchers have recognized the potential synergistic effects and complex interactions that choline salicylate may exhibit when combined with other substances^5,6^. Among these, *L*-glycine, an amino acid abundantly found in the body, has emerged as a promising candidate for interaction studies^7–9^. *L*-glycine, known for its involvement in central nervous system regulation, collagen synthesis, and digestive processes^10–12^, can potentially influence the behavior of choline salicylate in aqueous solution.

The thermodynamic properties of choline salicylate and *L*-glycine in aqueous solution are of great significance for formulating stable products and understanding their behavior in complex matrices. Parameters such as solubility, phase behavior, and miscibility hold pivotal importance in optimizing the formulation process^13,14^. Additionally, transport properties, including diffusion coefficients and permeability, play a crucial role in predicting the bioavailability and drug delivery of choline salicylate and *L*-glycine when administered through various routes^15,16^.

Furthermore, exploring the interactions between choline salicylate and *L*-glycine can shed light on potential synergistic effects and complex formulation^17^. Such interactions may have implications for the stability, solubility, and pharmacological properties of the substances^18^. Understanding the thermodynamics of these interactions is vital for predicting and optimizing the behavior of choline salicylate and *L*-glycine in pharmaceutical or therapeutic applications.

This study aims to investigate the thermodynamic and transport properties of aqueous choline salicylate in the presence of *L*-glycine. In this respect, the double active pharmaceutical ingredient in ionic liquid form, [Ch][Sal] was synthesized and the density, speed of sound, viscosity and electrical conductance of it in water and aqueous solutions of *L*-glycine have been measured at *T* = (288.15 to 318.15) K and at atmospheric pressure and the different thermophysical properties have been utilized to calculate. By elucidating the behavior of these substances in solution, this research contributes to a deeper understanding of their interactions. A comprehensive understanding of the thermodynamic and transport properties of aqueous choline salicylate in the presence of *L*-glycine is crucial for optimizing its formulation, enhancing bioavailability. This study bridges the gap in current knowledge and provides valuable insights into the behavior and interactions of these substances, paving the way for improved pharmaceutical preparations and therapeutic interventions.

## Materials and methods

### Materials

The detailed descriptions of the used materials including the supplier, CAS number, and purity are specified in Table 1.

**Table 1:** Specifications of the used chemicals in this work.

Chemical name	CAS No.	Provenance	Supplier country	Purity (In mass fraction)
*L*-glycine	56-40-6	Loba Chemie	India	0.99
Choline salicylate	2016-36-6	Synthesized	–	0.98
Choline chloride	67-48-1	Sigma Aldrich	USA	> 0.99
Sodium salicylate	54-21-7	Merck	Germany	≥ 0.995
Acetone	67-64-1	Merck	Germany	> 0.998
Dichloromethane	75-09-2	Merck	Germany	≥ 0.999

Some thermophysical properties of choline salicylate ([Ch][Sal]) are presented in Table 2. Double distilled deionized water with a specific conductivity less than 0.055 µS.cm^− 1^ was used to prepare solutions containing [Ch][Sal] in the presence of *L*-glycine.

**Table 2:** Density (*d*) and speed of sound (*u*) data for [Ch][Sal] at different temperatures.

*T* /K	10^− 3^ *d* / kg m^− 3^	*u */ m s^− 1^
288.15	1193.764	1900.25
298.15	1187.620	1873.48
308.15	1181.589	1848.59
318.15	1175.636	1824.48

The standard uncertainty for temperature and pressure were *u*(*T*) = 0.01 K and *u*(*P*) = 0.001 MPa. The relative standard uncertainties *u*_*r*_(*d*) = 0.03, and *u*_*r*_ (*u*) = 0.5.

### Synthesis of the API-IL

Choline salicylate ([Ch][Sal]) was synthesized via ion exchange by dissolving sodium salicylate in dried acetone, followed by the addition of choline chloride under stirring. The mixture was stirred at 298 K, then refluxed at 329 K under nitrogen for 72 h. Sodium chloride was removed by filtration, and the solvent was evaporated at 329 K under reduced pressure (0.7 kPa) for 4 h. Residual salts were removed by washing with cold, dry dichloromethane, followed by phase separation. The solvent was evaporated at 312 K under high vacuum (0.1 Pa) for 3 h, yielding a clear, yellowish liquid.

Purity was confirmed by ^1^H NMR (Bruker Avance 400) and FTIR (Bruker Tensor 27) (Figs S1 and S2), showing no impurities. Karl Fischer titration determined a water content of 0.009 wt%, which was accounted for in calculations. Silver nitrate titration and potentiometry verified the absence of chloride ions.

### Apparatus and methods

A high-precision analytical balance (AND, GF202, Japan) with an uncertainty of ± 1 × 10⁻⁷ kg was employed to prepare solutions in molal-base and the measured solution density was used to convert them to molar concentration in the case of transport properties correlation. The samples were stored in tightly sealed glass vials using parafilm. A vibrating tube density and speed of sound analyzer (Anton Paar DSA 5000) was used to measure the density (*d*) and speed of sound (*u*) of the solutions under atmospheric pressure at a temperature range of (288.15-318.15) K with ± 0.002 K. The instrument was calibrated using dry air and deionized water under atmospheric pressure^19,20^, and measurement uncertainties for density and speed of sound are given in the corresponding tables, respctively^21,22^.

Viscosity measurements were carried out using a digital viscometer (Lovis 2000 M, Anton Paar) based on the principle of a falling sphere within a capillary of fixed diameter. Two laser sensors positioned at the ends of the capillary detected the sphere, enabling the calculation of the time taken to traverse the distance. The viscometer automatically recorded the average time over three runs. A Peltier device integrated into the viscometer maintained the temperature with a precision of ± 0.01 K. The uncertainty of viscosity measurements is given in the corresponding table.

The electrical conductance of the solutions was measured with titration method using a digital conductometer (Metrohm 712) equipped with platinized electrodes and operating at a frequency of 1 MHz under a nitrogen atmosphere. The cell constant was 0.960 cm⁻¹ after calibration with 0.01 mol L^− 1^ KCl aqueous solution supplied by Metrohm. Approximately 100 mL of double-distilled deionized water, along with varying amounts of *L*-glycine, was placed and sealed in the cell, then the ionic liquid was added dropwise into the cell using a syringe^23^. Temperature control was achieved with a thermostatically regulated water bath circulating around the cell, ensuring stability of ± 0.02 K using a Julabo NP thermostat (Germany) with a temperature stability of ± 0.01 K. The uncertainty in specific conductivity measurements was estimated to be less than 0.5% that the values are given in the corresponding table.

Density functional theory (DFT) calculations were conducted using the GGA VWN-BP functional within the Dmol³ module of Biovia Materials Studio 2023, utilizing the DND 3.5 basis set as recommended by the software developer. COSMO results were obtained following geometry optimization and subsequent energy optimization to explore the hydration behavior of the studied ionic liquid and *L*-glycine, particularly in terms of solvation energy and dielectric properties^24–27^.

## Results and discussions

### Volumetric properties

The density of [Ch][Sal] in water and aqueous solutions of *L*-glycine with different concentration under atmospheric pressure at *T* = (288.15–31815) K with 10 K intervals are listed in Table 3.

**Table 3:** The density (*d*) and apparent molar volume (${V_\varphi }$) for choline salicylate in the aqueous solutions of *L*-glycine at *T*= (288.15 to 318.15) K and *P* = 0.0868 MPa.

^b^*m* /mol kg^− 1^	*d* / kg m^− 3^					10^6^ *V*_*φ*_ / m^3^ mol^− 1^			
	288.15 K	298.15 K	308.15 K	318.15 K		288.15 K	298.15 K	308.15 K	318.15 K
[Ch][Sal] + water									
0.0000	999.094	997.042	994.020	990.190		–	–	–	–
0.0282	1000.398	998.300	995.237	991.228		194.92	196.87	198.81	205.91
0.0601	1001.872	999.722	996.596	992.394		194.65	196.61	198.84	205.81
0.0870	1003.082	1000.836	997.709	993.366		194.83	197.40	199.10	205.80
0.1076	1004.009	1001.778	998.501	994.054		194.76	196.77	199.67	206.23
0.1441	1005.647	1003.374	999.891	995.280		194.68	196.56	200.32	206.59
0.1656	1006.580	1004.290	1000.925	996.210		194.75	196.54	199.15	205.35
0.1817	1007.292	1005.021	1001.648	997.006		194.71	196.26	198.73	204.01
[Ch][Sal] + water + *L*-glycine (*m*_*L−gly*_ = 0.0986 mol kg^− 1^)									
0.0000	1002.320	1000.192	996.487	993.243		–	–	–	–
0.0312	1003.724	1001.542	997.809	994.521		195.63	197.69	199.17	201.12
0.0597	1005.024	1002.775	999.026	995.691		195.12	197.48	198.80	200.87
0.0885	1006.309	1003.998	1000.201	996.886		195.08	197.49	199.13	200.46
0.1149	1007.525	1005.119	1001.364	997.975		194.63	197.40	198.42	200.23
0.1517	1009.084	1006.644	1002.918	999.463		195.04	197.46	198.17	200.12
0.1794	1010.268	1007.830	1004.133	1000.503		195.08	197.18	197.70	200.44
0.2100	1011.577	1009.065	1005.265	1001.708		195.04	197.25	198.29	200.35
[Ch][Sal] + water + *L*-glycine (*m*_*L−gly*_ = 0.1981 mol kg^− 1^)									
0.0000	1005.462	1003.261	1000.149	996.266		–	–	–	–
0.0346	1006.990	1004.698	1001.541	997.490		196.04	198.99	200.79	206.33
0.0546	1007.935	1005.568	1002.370	998.402		194.67	198.04	200.11	202.32
0.0906	1009.473	1006.898	1003.671	999.909		195.40	199.89	201.68	200.94
0.1060	1010.203	1007.685	1004.465	1000.478		194.83	198.17	199.69	201.32
0.1376	1011.571	1008.980	1005.747	1001.701		194.86	198.06	199.44	201.28
0.1753	1013.110	1010.502	1007.251	1003.015		195.34	198.04	199.34	202.04
0.2022	1014.292	1011.542	1008.153	1004.215		195.07	198.18	200.08	200.98
[Ch][Sal] + water + *L*-glycine (*m*_*L−gly*_ = 0.3014 mol kg^− 1^)									
0.0000	1008.594	1006.287	1002.930	999.017		–	–	–	–
0.0310	1009.924	1007.544	1004.087	1000.073		196.78	199.47	203.23	207.16
0.0588	1011.130	1008.663	1005.124	1001.024		196.31	199.37	203.01	206.88
0.0927	1012.575	1010.014	1006.348	1002.158		196.25	199.35	203.24	206.93
0.1133	1013.455	1010.840	1007.216	1002.834		196.11	199.19	202.11	206.97
0.1422	1014.701	1011.983	1008.262	1003.876		195.82	199.09	202.22	206.28
0.1820	1016.338	1013.553	1009.667	1005.146		195.90	198.92	202.42	206.51
0.2039	1017.240	1014.375	1010.447	1005.969		195.86	199.00	202.41	205.92

The standard uncertainty for temperature and pressure were *u*(*T*) = 0.01 K and u(*P*) = 0.001 MPa. The relative standard uncertainties, *u*_*r*_(*d*) = 0.03, *u*_*r*_($\:{V}_{\phi\:}$) = 0.3 $\:\times\:\:$10^− 6^, *u*_*r*_(*m*) = 0.002 and *u*_*r*_(*m*_*gly*_) = 0.005 where *m*_*gly*_ is the molal concentration of *L*-glycine in water.

**Fig. 1:**
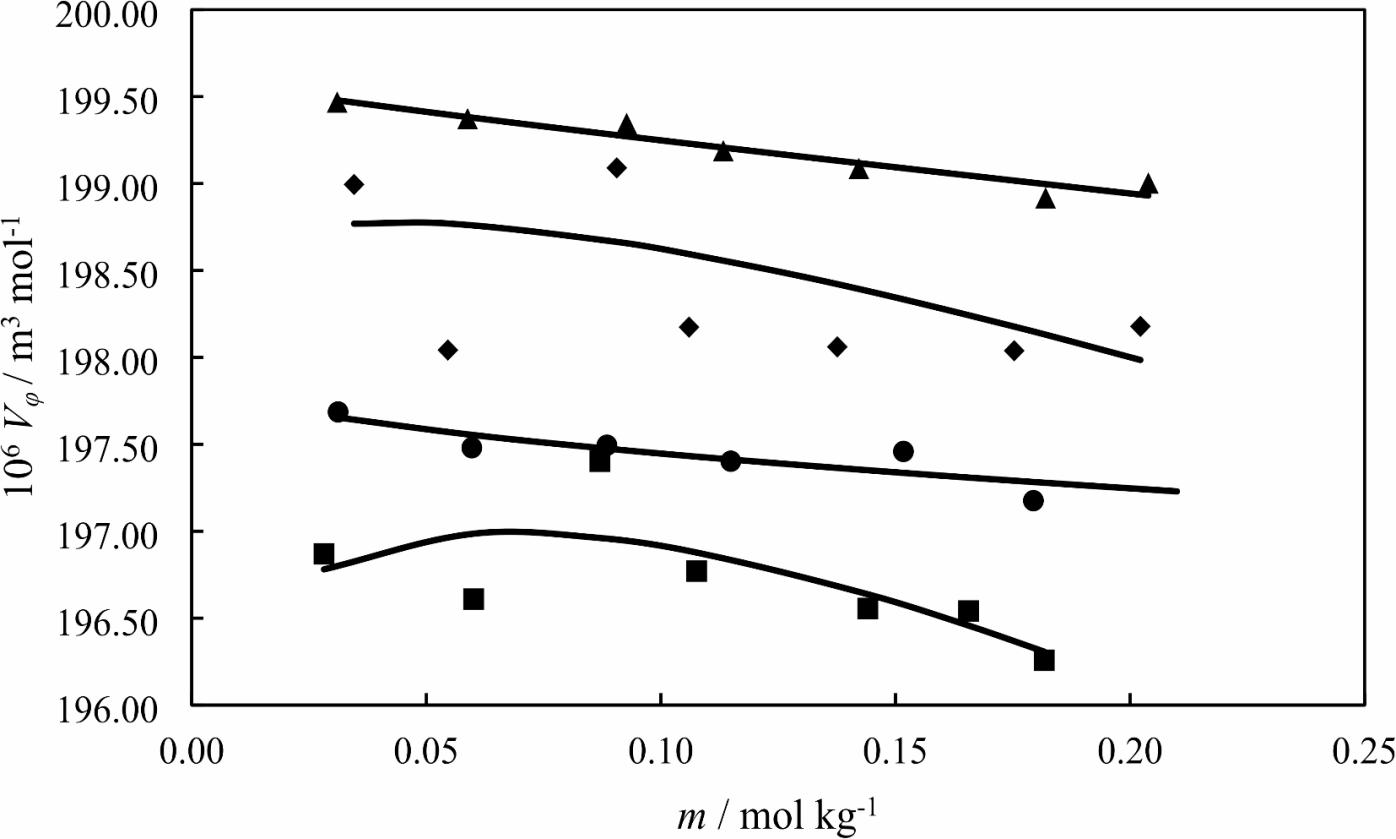
The apparent molar volumes of [Ch][Sal] as a function of its molality in the presence of different concentration of *L*-glycine in aqueous: (∎) 0.0000; (●) 0.1068; (♦) 0.2034; (▲) 0.3002 mol kg^− 1^ under atmospheric pressure at *T* = 298.15 K.

The density of aqueous *L*-glycine solutions are in good agreement with literature^28,29^. The density of the solutions are increased by addition of the [Ch][Sal] and *L*-glycine content and decrease with temperature raise. Apparent molar volumes (${V_\varphi }$) of [Ch][Sal] are calculate with Eq. (1) using the measured density of the solutions^20^:

1 $${V_\varphi }=\frac{M}{d} - \left[ {\frac{{(d - {d_0})}}{{md{d_0}}}} \right]$$

where, *m* and *M* are molality and molar mass of [Ch][Sal], respectively. Also, *d*_*0*_ and *d*, are the densities of the solvent (water or water + *L*-glycine) and solutions. The plot of apparent molar volumes of [Ch][Sal] as a function of its molality are shown in Fig. 1.

It is implied that the apparent molar volumes increase as the concentration of *L*-glycine increases. The standard partial molar volume, ${V_\varphi }^{0}$, is obtained by nonlinear regression of the apparent molar volume by following equation:^30^

2 $${V_\varphi }={V_\varphi }^{0}+{S_v}{m^{1/2}}+{b_v}m$$

where, ${V_\varphi }^{0}$ is the standard partial molar volume, *S*_*v*_ and *b*_*v*_ are empirical parameters of the equation. Since solute-solute interactions (cation-anion) at infinite dilution are negligible, important information on solute-solvent interactions could be analyzed by standard partial molar volumes. The values of ${V_\varphi }^{0}$, *S*_*v*_ and *b*_*v*_ together with the standards deviation of the ${V_\varphi }^{0}$ are reported in Table 4.

**Table 4:** The values of standard apparent molar volume, $V_{\varphi }^{0}$ ,experimental slop,${b_v}$, transfer volumes, $\:{\varDelta\:}_{tr}{V}_{\phi\:}^{0}$ standard apparent molar expansibility, $E_{\varphi }^{0}$, coefficient of thermal expansion, α, Hepler’s constant, ${({\partial ^2}V_{\varphi }^{0}/\partial {T^2})_P}$of [Ch][Sal] in water and in the aqueous solutions of *L*-glycine at *T* = (288.15 to 318.15) K and *P* = 0.0868 MPa.

*T*/ K	10^6^ ${V_\varphi }^{0}$/ m^3^ mol^− 1^	10^6^*S*_V_ / m^3^ mol^− 1^ kg^− 1/2^	10^6^*b*_V_ / m^3^·mol^− 1^ kg^− 1^	10^6^$\:\sigma\:\left({V}_{\phi\:}\right)$	10^6^$\:{\varDelta\:}_{tr}{V}_{\phi\:}^{0}$/ m^3^ mol^− 1^	10^6^$\:{(E}_{\phi\:}^{0})$/ m^3^ mol^− 1^ K^− 1^	10^3^$\:\alpha\:$/K^− 1^	10^6^$\:{(\partial\:}^{2}{V}_{\phi\:}^{0}$/$\:\partial\:{T}^{2}$)_*P*_ / m^3^ mol^− 1^ K^− 2^
[Ch][Sal] + water								
288.15	195.26 ± 0.00	− 2.96 ± 0.03	4.02 ± 0.05	0.07	–	− 0.25 ± − 0.01	− 1.28 ± 0.01	-0.03
298.15	195.31 ± 0.02	12.89 ± 0.11	− 24.78 ± 0.17	0.10	–	0.05 ± 0.00	0.26 ± 0.00	
308.15	195.73 ± 0.09	22.71 ± 0.21	− 34.34 ± 0.34	0.45	–	0.35 ± 0.01	1.79 ± 0.02	
318.15	201.80 ± 0.04	33.16 ± 0.26	− 61.30 ± 0.42	0.31	–	0.65 ± 0.02	3.23 ± 0.03	
[Ch][Sal] + water + *L*-glycine (*m*_*L−gly*_ = 0.0986 mol·kg^− 1^)								
288.15	197.83 ± 0.01	− 16.69 ± 0.05	23.59 ± 0.08	0.03	2.57	− 0.07 ± 0.00	− 0.33 ± 0.00	-0.02
298.15	197.91 ± 0.00	− 1.39 ± 0.03	− 0.18 ± 0.05	0.03	2.60	0.10 ± 0.00	0.52 ± 0.01	
308.15	199.92 ± 0.03	− 3.82 ± 0.11	− 1.02 ± 0.18	0.21	4.19	0.27 ± 0.01	1.35 ± 0.01	
318.15	203.35 ± 0.01	− 15.76 ± 0.05	20.18 ± 0.07	0.11	1.55	0.44 ± 0.01	2.15 ± 0.02	
[Ch][Sal] + water + *L*-glycine (*m*_*L−gly*_ = 0.1981 mol·kg^− 1^)								
288.15	198.75 ± 0.02	− 22.31 ± 0.16	32.35 ± 0.25	0.02	3.49	− 0.80 ± − 0.02	− 4.03 ± − 0.04	-0.10
298.15	198.16 ± 0.04	5.84 ± 0.28	− 13.86 ± 0.43	0.30	2.85	0.20 ± 0.01	0.99 ± 0.01	
308.15	201.46 ± 0.22	− 3.48 ± 0.31	− 1.43 ± 0.48	0.06	5.73	1.19 ± 0.04	5.92 ± 0.06	
318.15	220.82 ± 0.05	− 109.72 ± 0.34	150.17 ± 0.53	0.04	19.02	2.19 ± 0.07	9.92 ± 0.10	
[Ch][Sal] + water + *L*-glycine (*m*_*L−gly*_ = 0.3014 mol·kg^− 1^)								
288.15	198.12 ± 0.00	− 9.52 ± 0.03	9.96 ± 0.05	0.08	2.86	0.27 ± 0.01	1.39 ± 0.01	0.00
298.15	199.63 ± 0.00	− 0.39 ± 0.02	− 2.55 ± 0.03	0.04	4.32	0.30 ± 0.01	1.48 ± 0.01	
308.15	204.71 ± 0.03	− 9.49 ± 0.12	9.30 ± 0.19	0.02	8.98	0.32 ± 0.01	1.54 ± 0.02	
318.15	206.62 ± 0.01	5.18 ± 0.08	− 14.20 ± 0.12	0.20	4.82	0.34 ± 0.01	1.63 ± 0.02	

The standard uncertainty for temperature and pressure were *u*(*T*) = 0.01 K and *u*(*P*) = 0.001 MPa.

The results show that the values of ${V_\varphi }^{0}$as a criterion of the solute-solvent interactions are positive and increase with an increase in both *L*-glycine concentration and temperatures. The larger values at high temperature are probably refers to the release of the solvent molecules around [Ch][Sal] into the bulk of the solvent^20^. The standard partial molar volume of the [Ch][Sal] can be calculated from standard partial molar volume of [Ch][Cl] and [Na][Sal] at 298.15 K. Based on the literature reports, standard partial molar volumes of choline chloride, and sodium salicylate are 10^6^${V_\varphi }^{0}$ ([Ch][Cl] ) = 122.76 m^3^ mol^− 1^^31^, 10^6^${V_\varphi }^{0}$ ([Na][Sal]) = 93.75 m^3^ mol^− 1^^32^ while chloride and sodium ions are 10^6^${V_\varphi }^{0}$ ([Cl]^−^) = 17.83 m^3^ mol^− 1^ and 10^6^${V_\varphi }^{0}$ (Na^+^) = -1.21 m^3^ mol^−1^^33^ at *T* = 298.15 K. Accordingly, the calculated standard partial molar volume of choline salicylate is 10^6^${V_\varphi }^{0}$ ([Ch][Sal]) = 199.89 m^3^mol^− 1^ that are in good agreement with the values obtained from Eq. (2) and the numerical values are available in Table 4. The temperature dependency of ${V_\varphi }^{0}$ can be correlated with following equation:

3 $${V_\varphi }^{0}=A+BT+C{T^2}$$

where *A*,* B* and *C* are empirical parameters calculated by the least-square fitting of the standard partial molar volume as function of temperature^34^. The derivative of standard partial molar volume ${V_\varphi }^{0}$ from Eq. 3 as function of temperature at constant pressure are defined as the standard apparent molar expansibilities $E_{\varphi }^{0}$ which are given in Table 4. The positive $E_{\varphi }^{0}$ values increase with *L*-glycine concentration increment and intensifies at higher temperatures. This indicate at higher temperature; some of water molecules as hydration layer of [Ch][Sal] are released from the solvation layers with the presence of *L*-glycine molecules. Positive expansibility is a characteristic property of aqueous solutions of hydrophobic hydration. This phenomenon would intensify the solution volume increasing rapidly in the presence *L*-glycine than the pure water^20,34^. The apparent thermal expansion coefficient,$\alpha$, were calculated using Eq. (4):^20,30^

4 $$\alpha =\frac{{E_{\varphi }^{0}}}{{V_{\varphi }^{0}}}$$

The $\alpha$ values of [Ch][Sal] are given in Table 4. This parameter is basically a balance to evaluate the reaction of the solutions bulk to temperature variations that according to the values given in Table 4, this variable decreases with increasing of temperature that implies the volume increment would be intensified in the presence of *L*-glycine at higher temperature. The sign of the second derivative of $V_{\varphi }^{0}$with respect to temperature are defined as Hepler’s constant as a qualitative interpretation about structure maker or structure breaker behavior of solute in the bulk of the solutions that are defined by following equation:^20,30^

5 $${-\left( {{\partial ^2}V_{\varphi }^{0}/\partial {T^2}} \right)_\text{P}}=-2C$$

The values of ${({\partial ^2}{V_\varphi }^{0}/\partial {T^2})_p}$ for studied systems are given in Table 4. Negative value are associate with a structure-breaking behavior of the [Ch][Sal] in the aqueous solutions of the aqueous *L*-glycine solutions^22,30^. It is noticed that the values of ${({\partial ^2}{V_\varphi }^{0}/\partial {T^2})_p}$are negative and become zero with an increase of *L*-glycine concentration which implies that [Ch][Sal] essentially acts as a structure breaker. The partial molar volume of transfer, ${\Delta _{tr}}\mathop V\nolimits_{\varphi }^{0}$, of [Ch][Sal] from water to the aqueous *L*-glycine solutions have been calculated by the following equation:^20,35^

6 $${\Delta _{tr}}\mathop V\nolimits_{\varphi }^{0} =\mathop V\nolimits_{\varphi }^{0} ([Ch][Sal]+L - {\text{glycine+water}}) - \mathop V\nolimits_{\varphi }^{0} ([Ch][Sal]+{\text{water}})$$

The values of ${\Delta _{tr}}\mathop V\nolimits_{\varphi }^{0}$ for the solutions are given in Table 4. The ${\Delta _{tr}}\mathop V\nolimits_{\varphi }^{0}$ values are positive at all temperatures and increase with increasing in concentration of *L*-glycine. The type of interactions possible between solute ([Ch][Sal] ) and co-solute (*L*-glycine) in the solutions ( [Ch][Sal] + *L*-glycine + water) are: (i), ion–hydrophilic and hydrophilic–hydrophilic interactions (between ions of [Ch][Sal] with *L*-glycine polar groups) such as: COO^*−*^ and N^+^ interactions with water molecules and with each other, (ii) ion- hydrophobic interactions between ions of [Ch][Sal] and non-polar groups of amino acid, (iii) hydrophobic-hydrophobic interactions (between alkyl groups of [Ch][Sal] and hydrophobic groups of *L*-glycine), and (iv) hydrophilic–hydrophobic interactions between the cation and anion parts of the [Ch][Sal] and hydrophobic groups of *L*-glycine. Based on co-sphere overlap model^20,35,36^ that are applicable on ${\Delta _{tr}}\mathop V\nolimits_{\varphi }^{0}$ that eliminates water effect in the existing interactions between the other components of the solution, two types of interactions including ion–hydrophilic and hydrophilic–hydrophilic have positive effects on ${\Delta _{tr}}\mathop V\nolimits_{\varphi }^{0}$ values whereas, other types result in negative transfer volumes. The positive values of ${\Delta _{tr}}\mathop V\nolimits_{\varphi }^{0}$ values in the studied systems express that hydrophilic interactions overcome to the other types of interactions and this trend becomes stronger with increasing in concentration of *L*-glycine^20,35^. As a result of these interactions, the water molecules are allowed to enter into bulk of the solution and this is the reason for the observed positive transfer volumes.

###  Compressibility results

The measured speeds of sound, *u*, of the studied solutions at different temperatures are shown in Table 5.

**Table 5:** The speed of sound (*u*) of the solutions and apparent molar isentropic compressibility (${\kappa _\varphi }$) of [Ch][Sal] in the presence of different concentration of *L*-glycine in aqueous solution under atmospheric pressure and at *T* = (288.15 to 318.15) K.

*m* / mol kg^− 1^	*u* (m s^− 1^)				10^14^${\kappa _\varphi }$_(m_^3^_mol_^−1^_Pa_^−1^_)_			
	288.15 K	298.15 K	308.15 K	318.15 K	288.15 K	298.15 K	308.15 K	318.15 K
[Ch][Sal] + water								
0.0000	1466.76	1496.76	1519.51	1535.94	**–**	**–**	**–**	**–**
0.0282	1470.65	1500.24	1522.57	1538.48	− 1.86	− 0.61	0.48	2.12
0.0601	1475.02	1504.14	1526.13	1541.61	− 1.85	− 0.60	0.38	1.86
0.0870	1478.64	1507.36	1528.99	1544.11	− 1.77	− 0.48	0.47	1.88
0.1076	1481.37	1509.67	1531.15	1545.91	− 1.74	− 0.43	0.56	1.97
0.1441	1486.24	1513.93	1534.65	1549.22	− 1.70	− 0.40	0.79	2.02
0.1656	1488.89	1516.25	1536.80	1550.94	− 1.60	− 0.32	0.72	2.00
0.1817	1491.05	1518.12	1538.22	1552.18	− 1.60	− 0.34	0.76	1.95
[Ch][Sal] + water + *L*-glycine (*m*_*L−gly*_ = 0.0986 mol kg^− 1^)								
0.0000	1472.10	1501.93	1524.38	1540.69	**–**	**–**	**–**	**–**
0.0312	1476.58	1505.82	1527.87	1543.60	− 2.03	− 0.53	0.37	1.57
0.0597	1480.40	1509.26	1530.73	1546.33	− 1.79	− 0.44	0.65	1.48
0.0885	1484.29	1512.80	1533.93	1548.80	− 1.71	− 0.45	0.59	1.60
0.1149	1487.89	1515.67	1536.66	1551.12	− 1.73	− 0.27	0.58	1.62
0.1517	1492.60	1519.88	1540.11	1554.58	− 1.56	− 0.19	0.73	1.56
0.1794	1496.01	1522.85	1542.73	1556.66	− 1.44	− 0.12	0.76	1.72
0.2100	1499.87	1526.09	1545.57	1559.72	− 1.38	− 0.03	0.88	1.61
[Ch][Sal] + water + *L*-glycine (*m*_*L−gly*_ = 0.1981 mol kg^− 1^)								
0.0000	1477.64	1507.08	1529.25	1545.42	**–**	**–**	**–**	**–**
0.0346	1482.45	1511.39	1533.22	1549.28	− 1.61	− 0.32	0.47	1.12
0.0546	1485.17	1513.78	1535.29	1550.78	− 1.60	− 0.30	0.53	1.36
0.0906	1490.03	1518.37	1539.37	1553.86	− 1.54	− 0.22	0.64	1.62
0.1060	1492.00	1519.77	1540.89	1555.12	− 1.51	− 0.17	0.69	1.69
0.1376	1496.26	1523.42	1543.80	1558.09	− 1.43	− 0.07	0.78	1.79
0.1753	1500.89	1527.29	1547.25	1560.93	− 1.33	0.07	0.90	1.85
0.2022	1504.37	1530.21	1550.17	1563.16	− 1.25	0.18	0.98	1.87
[Ch][Sal] + water + *L*-glycine (*m*_*L−gly*_ = 0.3014 mol kg^− 1^)								
0.0000	1482.56	1511.69	1533.58	1549.24	**–**	**–**	**–**	**–**
0.0310	1486.65	1515.58	1536.87	1552.11	− 1.00	− 0.26	1.17	2.20
0.0588	1490.03	1518.78	1539.73	1554.65	− 0.76	0.00	1.23	2.20
0.0927	1494.55	1522.76	1543.08	1558.39	− 0.90	0.07	1.36	1.84
0.1133	1497.10	1524.81	1544.66	1559.95	− 0.85	0.25	1.51	2.06
0.1422	1500.47	1527.73	1547.42	1561.77	− 0.74	0.41	1.54	2.33
0.1820	1504.61	1531.35	1550.84	1564.92	− 0.45	0.65	1.68	2.45
0.2039	1506.87	1533.59	1552.43	1565.98	− 0.34	0.69	1.80	2.61

The standard uncertainty for temperature and pressure were *u*(*T*) = 0.01 K and u(P) = 0.001 MPa. The relative standard uncertainties, *u*_*r*_(*u*) = 0.5, *u*_*r*_($\:{\kappa\:}_{\phi\:}$) = 0.2 $\:\times\:\:$10^− 14^, *u*_*r*_(*m*) = 0.002 and *u*_*r*_(*m*_*gly*_) = 0.005 where *m*_*gly*_ is the molal concentration of *L*-glycine in water.

The speed of sound of aqueous *L*-glycine solutions are in good agreement with the literature^37^. The Laplace–Newton’s Eq. (7) was used to calculate the isentropic compressibility of the solutions:

7 $$\kappa _{s} - \frac{1}{{du^{2} }}$$

**Fig. 2:**
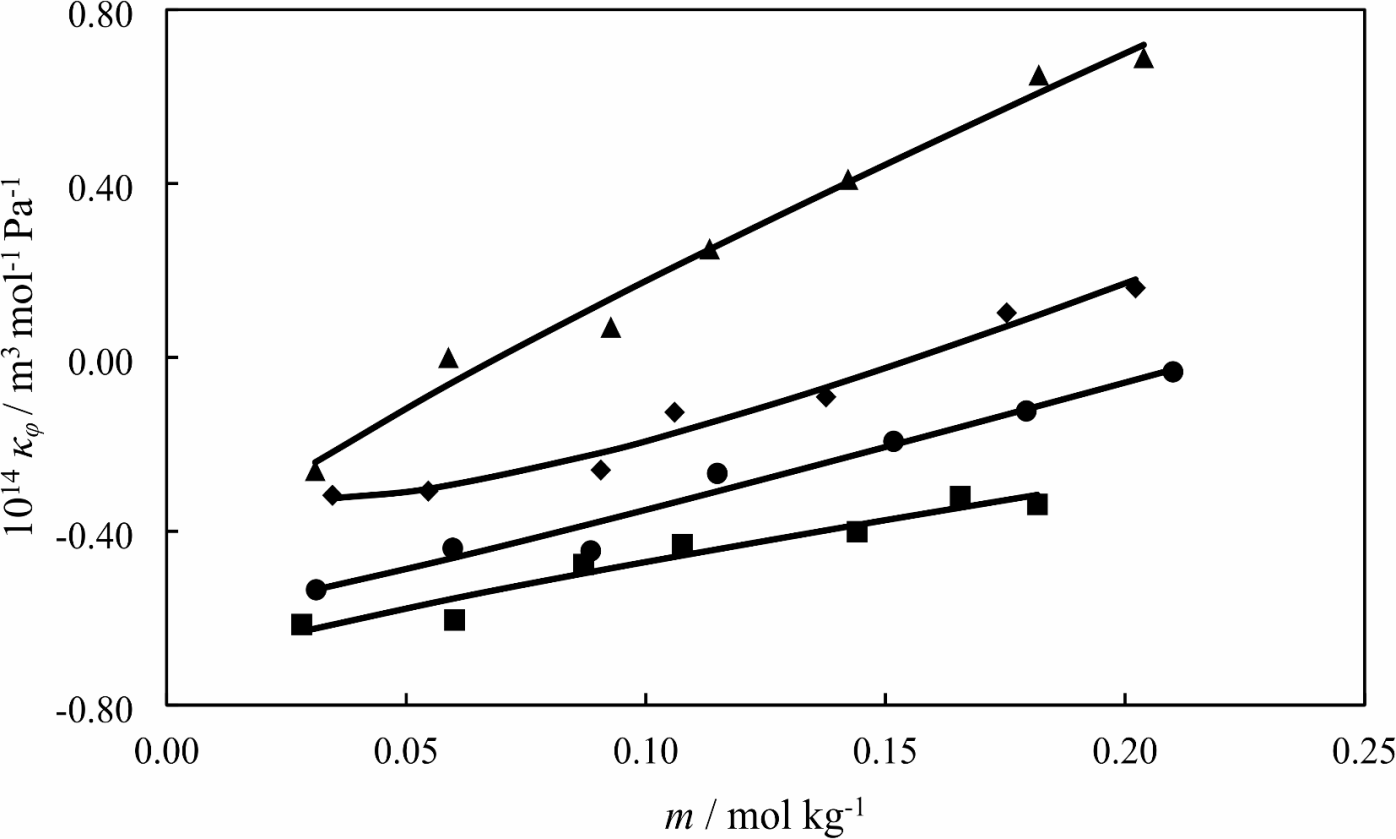
The apparent molar isentropic compressibility values of [Ch][Sal] as a function its molality in the presence of different concentration of *L*-glycine: (∎) 0.0000; (●) 0.1068; (♦) 0.2034; (▲) 0.3002 mol kg^− 1^ under atmospheric pressure and at *T* = 298.15 K.

The isentropic compressibility (${\kappa _s}$) is defined by the participation of two parts ${\kappa _{s1}}$(solvent intrinsic) is due to the compression of the solvent (water or aqueous solutions of *L*-glycine), and ${\kappa _{s2}}$(solute intrinsic, [Ch][Sal]) is due to the compression layer of solute molecules due to the influence of solvent molecules into the empty space around the solute([Ch][Sal])^35^. In this respect, the apparent molar isentropic compressibility (${\kappa _\varphi }$) of [Ch][Sal] is calculated with the Eq. (8):

8 $${\kappa _\varphi }=\frac{{({\kappa _s}{d_0} - {\kappa _{s0}}d)}}{{md{d_0}}}+\frac{{{\kappa _s}M}}{d}$$

where, ${\kappa _{s0}}$, and ${\kappa _s}$ are isentropic compressibility values of the solvent and solutions, respectively and the other symbols are same as previously defined. The calculated ${\kappa _\varphi }$values are given in Table 5. Also, the ${\kappa _\varphi }$ values of [Ch][Sal] in the aqueous solutions in the presence of different concentration of *L*-glycine under atmospheric pressure at 298.15 K are shown in Fig. 2.

As presented in Fig. 2, the ${\kappa _\varphi }$ values are increased with the concentration of *L*-glycine at higher temperatures. The variation of ${\kappa _\varphi }$ with molality of [Ch][Sal] are correlated with the Eq. (9):

9 $${\kappa _\varphi }=\kappa _{\varphi }^{0}+{S_k}{m^{1/2}}+{b_k}m$$

where,$\kappa _{\varphi }^{0}$ is partial molar isentropic compressibility, ${S_k}$ and ${b_k}$ are the empirical parameters of this equation. The calculated values of $\kappa _{\varphi }^{0}$, ${S_k}$ and ${b_k}$ are given in Table 6 with corresponding standard deviations.

**Table 6:** The values of $\kappa _{\varphi }^{0}$,${S_k}$ and ${b_k}$ parameters, Prtial molar isentropic compressibility of transfer, (${\Delta _{tr}}\kappa _{\varphi }^{0}$), for [Ch][Sal] in water and in the aqueous solutions of *L*-glycine under atmospheric pressure at *T* = (288.15 to 318.15) K.

*T*/K	10^14^$\:{}\kappa_{\phi\:}^{0}$ /m^3^ mol^− 1^ Pa^− 1^	10^14^*S*_k_ /m^3^ mol^− 2^ kg Pa^− 1^	10^14^*b*_k_ / m^3^ mol^− 2^ kg Pa^− 1^	10^14^${\Delta _{tr}}\kappa _{\varphi }^{0}$/ m^3^ mol^− 1^ Pa^− 1^	10^14^σ(κ_φ_)
[Ch][Sal] + water					
288.15	− 1.80 ± 0.02	− 0.91 ± 0.17	3.30 ± 0.34	–	0.01
298.15	− 0.74 ± 0.00	0.47 ± 0.02	1.26 ± 0.11	–	0.05
308.15	0.80 ± 0.00	− 3.40 ± 0.01	8.06 ± 0.05	–	0.05
318.15	2.71 ± 0.00	− 5.17 ± 0.04	8.30 ± 0.21	–	0.07
[Ch][Sal] + water + *L*-glycine (*m*_*L−gly*_ = 0.0986 mol kg^− 1^)					
288.15	− 2.36 ± 0.09	2.00 ± 0.72	0.28 ± 1.40	− 0.56	0.03
298.15	− 0.58 ± 0.00	− 0.36 ± 0.01	3.41 ± 0.03	0.16	0.04
308.15	0.23 ± 0.00	1.03 ± 0.01	0.68 ± 0.04	− 0.57	0.21
318.15	1.57 ± 0.00	− 0.32 ± 0.00	1.12 ± 0.01	− 1.14	0.11
[Ch][Sal] + water + *L*-glycine (*m*_*L−gly*_ = 0.1981 mol kg^− 1^)					
288.15	− 1.43 ± 0.05	− 1.98 ± 0.40	5.31 ± 0.79	0.37	0.29
298.15	− 0.12 ± 0.00	− 2.36 ± 0.00	6.72 ± 0.02	0.62	0.08
308.15	0.37 ± 0.00	− 0.06 ± 0.01	3.14 ± 0.05	− 0.43	0.60
318.15	− 0.22 ± 0.00	9.01 ± 0.04	− 9.70 ± 0.21	− 2.93	0.35
[Ch][Sal] + water + *L*-glycine (*m*_*L−gly*_ = 0.3014 mol kg^− 1^)					
288.15	− 0.45 ± 0.16	− 4.54 ± 1.31	10.45 ± 2.54	1.35	0.10
298.15	− 0.55 ± 0.00	1.11 ± 0.02	3.78 ± 0.12	0.19	0.04
308.15	1.08 ± 0.00	− 0.29 ± 0.01	4.10 ± 0.07	0.28	0.07
318.15	3.68 ± 0.00	− 11.83 ± 0.03	21.05 ± 0.17	0.97	0.15

The standard uncertainty for temperature and pressure were *u*(*T*) = 0.01 K and *u*(*P*) = 0.001 MPa.

The small ${b_k}$ values rather than $\kappa _{\varphi }^{0}$ indicates negligible solute-solute interactions compared to the solute–solvent interactions. The $\kappa _{\varphi }^{0}$ values increase from negative values towards positive values by increasing the *L*-glycine concentration at high temperatures. The more negative $\kappa _{\varphi }^{0}$ values at low temperatures are related to the strong attractive interactions due to the hydration of solute. At higher temperatures water molecules are released into bulk, thereby making the medium more compressible. Increasing *L*-glycine concentration led to form of ion-pairs cause to suppressed the electrostatic interactions between [Ch][Sal] and water molecules that are responsible for the higher compressibility^20,35^. Partial molar isentropic compressibility of transfer, ${\Delta _{tr}}\kappa _{\varphi }^{0}$, for water and the aqueous solutions of *L*-glycine have been calculated using the following equation:

10 $${\Delta _{tr}}\kappa _{\varphi }^{0}=\kappa _{\varphi }^{0}(\left[ {{\text{Ch}}} \right]\left[ {{\text{Sal}}} \right]\,+\,L - {\text{glycine}}\,+\,{\text{water}})--\kappa _{\varphi }^{0}(\left[ {{\text{Ch}}} \right]\left[ {{\text{Sal}}} \right]\,+\,{\text{water}})$$

The values of ${\Delta _{tr}}\mathop \kappa \nolimits_{\varphi }^{0}$ for studied solutions are represented in Table 6. The sign of${\Delta _{tra}}\mathop \kappa \nolimits_{\varphi }^{0}$ is positive in the studied system and these values increase with a rise in the concentration of *L*-glycine. Positive values of $\mathop \kappa \nolimits_{\varphi }^{0}$ for [Ch][Sal] illustrates the dominance of the head charged groups N^+^ and COO^–^ o8f *L*-glycine with the ions of [Ch][Sal] which increase with a rise in the concentration of *L*-glycine. This behavior which observed for partial molar isentropic compressibility of transfer, are in good agreement with volumetric results and supports them.

### Viscosity results

**Fig. 3:**
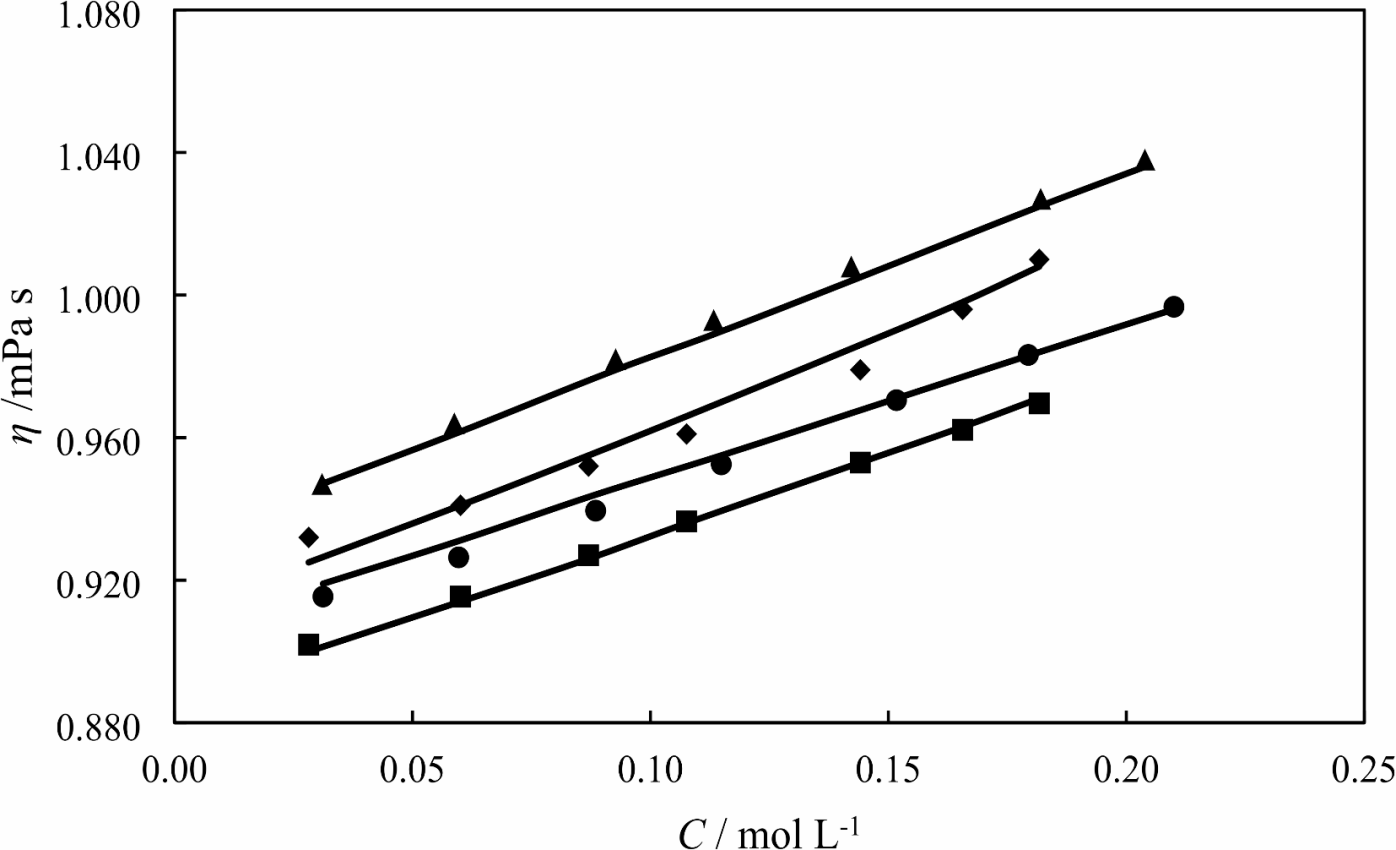
The variation of solutions viscosity with [Ch][Sal] molarity in the presence of different concentration of *L*-glycine: (∎) 0.0000; (●) 0.1068; (♦) 0.2034; (▲) 0.3002 mol kg^− 1^ under atmospheric pressure and at *T* = 298.15 K.

The viscosity of the solutions containing [Ch][Sal] in water in the presence of different concentration of *L*-glycine are given in Table 7. The measured viscosities, *η* of aqueous *L*-glycine solutions are in good agreement with those values of reported in the reference^29^. The variations of viscosity for the solutions at *T* = 298.15 K are plotted in Fig. 3.

**Table 7:** The viscosity (*η*) data of the solutions ([ch][sal] + water), and ([Ch][Sal] + *L*-glycine + water) at *T* = (288.15 to 318.15) K.

*m* / mol kg^− 1^	*η* / mPa s			
	288.15 K	298.15 K	308.15 K	318.15 K
[Ch][Sal] + water				
0.0000	1.126	0.889	0.717	0.597
0.0282	1.149	0.902	0.723	0.610
0.0601	1.165	0.915	0.735	0.621
0.0870	1.179	0.927	0.745	0.631
0.1076	1.191	0.937	0.753	0.638
0.1441	1.212	0.953	0.766	0.651
0.1656	1.224	0.962	0.774	0.659
0.1817	1.233	0.970	0.782	0.678
[Ch][Sal] + water + *L*-glycine (*m*_*L−gly*_ = 0.0986 mol kg^− 1^)				
0.0000	1.143	0.903	0.731	0.616
0.0312	1.168	0.915	0.737	0.616
0.0597	1.182	0.926	0.750	0.623
0.0885	1.198	0.940	0.761	0.632
0.1149	1.215	0.953	0.773	0.639
0.1517	1.236	0.971	0.785	0.650
0.1794	1.254	0.983	0.793	0.661
0.2100	1.271	0.997	0.805	0.671
[Ch][Sal] + water + *L*-glycine (*m*_*L−gly*_ = 0.1981 mol kg^− 1^)				
0.0000	1.160	0.916	0.753	0.629
0.0282	1.189	0.932	0.762	0.632
0.0601	1.209	0.941	0.769	0.638
0.0870	1.223	0.952	0.781	0.646
0.1076	1.234	0.961	0.789	0.652
0.1441	1.260	0.979	0.803	0.664
0.1656	1.277	0.996	0.815	0.674
0.1817	1.291	1.010	0.824	0.683
[Ch][Sal] + water + *L*-glycine (*m*_*L−gly*_ = 0.3014 mol kg^− 1^)				
0.0000	1.178	0.929	0.733	0.633
0.0310	1.218	0.947	0.765	0.640
0.0588	1.237	0.964	0.778	0.647
0.0927	1.257	0.982	0.792	0.660
0.1133	1.272	0.993	0.801	0.667
0.1422	1.295	1.008	0.813	0.678
0.1820	1.324	1.027	0.827	0.691
0.2039	1.340	1.038	0.836	0.699

The standard uncertainty for temperature and pressure were *u*(*T*) = 0.1 K and *u*(*P*) = 0.001 MPa. The relative standard uncertainties, *u*_*r*_(*η*) = 0.01, *u*_*r*_(*m*) = 0.002 and *u*_*r*_(*m*_*gly*_) = 0.005 where *m*_*gly*_ is the molal concentration of *L*-glycine in water.

The addition of *L*-glycine to the solutions increases viscosities and at a certain molal concentration of *L*-glycine. Higher temperatures cause to weaken solute and solvent interactions, and then solvent molecules freely enter to the empty spaces in solution at low viscosity of the solution^38^. Variation of relative viscosity,$(\eta /{\eta _0})$of the solutions with molar concentration of [Ch][Sal] in the presence of different concentration of *L*-glycine has been correlated by empirical Jones–Dole equation:^21^

11 $$\frac{\eta }{{{\eta _0}}}=1+A{C^{1/2}}+BC$$

where$\eta$and ${\eta _0}$ are the viscosities of solutions and solvent, respectively and *C* is the molar concentration of [Ch][Sal] in the solution. The symbol *A* stands for the Falkenhagen coefficient which indicates ion-pair electrostatic interaction and *B* is viscosity *B*-coefficient that is used to determine the solute-solvent interactions^21,30^. The calculated *A*, viscosity *B*-coefficients and the corresponding standard deviations, $\:\sigma\:\left(\eta\:\right)$ for the studied solutions are listed in Table 8.

**Table 8:** The values of empirical Falkenhagen coefficient (*A*), viscosity *B*-coefficients, free energy of activation per mole of solvent, ($\:\varDelta\:{\mu\:}_{1}^{{0}^{}}$), and the solute, ($\:\varDelta\:{\mu\:}_{2}^{{0}^{}}$), and viscosity *B*-coefficients of transfer ($\:{\varDelta\:}_{tr}B$) for [Ch][Sal] in water and the aqueous solutions of *L*-glycine at *T* = (288.15 to 318.15) K.

*T*/K	*A* / dm^3/2^ mol^− 1/2^	*B */dm^3^·mol^− 1^	10^− 3^($\:\varDelta\:{\mu\:}_{1}^{{0}^{}}$)/kJ·mol^− 1^	($\:\varDelta\:{\mu\:}_{2}^{{0}^{}}$)/kJ·mol^− 1^	$\:{\varDelta\:}_{tr}B$ /dm^3^mol^− 1^	σ(η)
[Ch][Sal] + water						
288.15	− 0.06 ± 0.00	0.75 ± 0.01	–	–	–	0.01
298.15	− 0.02 ± 0.00	0.55 ± 0.01	–	–	–	0.02
308.15	− 0.01 ± 0.00	0.53 ± 0.01	–	–	–	0.01
318.15	0.07 ± 0.00	0.45 ± 0.00	–	–	–	0.03
[Ch][Sal] + water + *L*-glycine (*m*_*L−gly*_ = 0.0986 mol kg^− 1^)						
288.15	0.12 ± 0.01	0.24 ± 0.01	9.51 ± 0.00	62.39 ± 0.06	− 0.51	0.01
298.15	0.02 ± 0.00	0.44 ± 0.01	9.27 ± 0.00	64.65 ± 0.03	− 0.11	0.02
308.15	0.05 ± 0.00	0.35 ± 0.00	9.04 ± 0.00	66.78 ± 0.01	− 0.18	0.02
318.15	− 0.07 ± 0.00	0.56 ± 0.01	8.89 ± 0.00	69.09 ± 0.04	0.11	0.01
[Ch][Sal] + water + *L*-glycine (*m*_*L−gly*_ = 0.1981 mol kg^− 1^)						
288.15	− 0.02 ± 0.00	0.63 ± 0.01	9.54 ± 0.06	62.68 ± 0.02	− 0.12	0.02
298.15	− 0.05 ± 0.00	0.67 ± 0.00	9.29 ± 0.05	64.94 ± 0.01	0.12	0.01
308.15	− 0.13 ± 0.00	0.80 ± 0.01	9.11 ± 0.06	67.16 ± 0.02	0.27	0.01
318.15	− 0.17 ± 0.00	0.87 ± 0.01	8.94 ± 0.05	69.43 ± 0.01	0.42	0.01
[Ch][Sal] + water + *L*-glycine (*m*_*L−gly*_ = 0.3014 mol kg^− 1^)						
288.15	0.25 ± 0.01	− 0.11 ± 0.01	9.57 ± 0.02	62.81 ± 0.05	− 0.86	0.03
298.15	0.02 ± 0.00	0.52 ± 0.01	9.32 ± 0.09	65.07 ± 0.02	− 0.03	0.02
308.15	0.25 ± 0.01	0.10 ± 0.00	9.03 ± 0.01	67.17 ± 0.04	− 0.43	0.02
318.15	0.02 ± 0.00	0.44 ± 0.01	8.95 ± 0.08	69.53 ± 0.02	− 0.01	0.01

The standard uncertainty for temperature and pressure were *u*(*T*) = 0.1 K and *u*(*P*) = 0.001 MPa.

According to Table 8, the obtained viscosity *B*-coefficients are positive which suggests the presence of strong interactions between *L*-glycine and [Ch][Sal]. The sign of viscosity *B*-coefficient derivative with respect temperature i.e. d*B/*d*T* predicts the solute ability to act as structure maker or breaker in a special solvent^39^. The d*B/*d*T* values of [Ch][Sal] are positive that suggests [Ch][Sal] act as structure breaker in the studied solutions. The viscosity *B*-coefficients of transfer, ${\Delta _{tra}}B$ from water to the aqueous *L*-glycine solutions have been obtained as follows:

12 $${\Delta _{tr}}B=B([Ch][Sal]+L - glycine+water) - B([Ch][Sal]+water)$$

The calculated values of ${\Delta _{tr}}B$ for studied solutions are listed in Table 8. The viscosity *B*-coefficients for the solutions containing *L*-glycine are lower than in water. Positive ${\Delta _{tr}}B$, values that decreases with addition of *L*-glycine show weakening of the interaction between [Ch][Sal] and the solvent. This phenomenon previously attributed to the water molecules release into the bulk of the solutions with the presence of *L*-glycine. The viscosity data have also been analyzed with transition state theory for relative viscosity by Feakins and co-workers^40,41^. The viscosity *B*-coefficient in terms of this theory is given by the following Eq. (13):

13 $$B=(\bar {V}_{1}^{0} - \bar {V}_{2}^{0})+\bar {V}_{1}^{0}\left(\frac{{\Delta \mu _{2}^{{0*}} - \Delta \mu _{1}^{{0*}}}}{{RT}}\right)$$

where, $\left( {\bar{V}_{1}^{0} = \sum {\frac{{x_{i} M_{i} }}{d }} } \right)$ is the molar volume of solvent while the symbol $(\bar {V}_{2}^{0}=V_{\varphi }^{0})$ represents the standard partial molar volume of the solute. The term $\bar {V}_{1}^{0}$ is the molar volume of the pure solvent. The terms *x*_*i*_ and ${M_i}$ denote the mole fraction and molar mass of solvent, *d* is the density of solvent (*L*-glycine + water). The solvent and solute molar free energy of activation $\Delta \mu _{1}^{{0*}}$and $\Delta \mu _{2}^{{0*}}$ are given in Table 8 that were calculated with Eyring’s model:

14 $$\Delta \mu _{1}^{{0*}}=\Delta G_{1}^{{0*}}=RT\ln \frac{{{\eta _1}V_{1}^{0}}}{{h{N_A}}}$$

15 $$\Delta \mu _{2}^{{0*}}=\Delta G_{2}^{{0*}}=\frac{{RT}}{{V_{1}^{0}}}[B - (\bar {V}_{1}^{0} - \bar {V}_{2}^{0})]$$

where,* h* and${N_A}$ symbols stand for the Planck constant and the Avogadro number, respectively. The large positive values of$\Delta \mu _{2}^{{0*}}$ than $\Delta \mu _{1}^{{0*}}$lead to the lower tendencies to form transition state due to strong solute-solvent interactions between [Ch][Sal] and *L*-glycine molecules. The values of$\Delta \mu _{2}^{{0*}}$, increase with rising of temperature and the concentration of *L*-glycine that first one indicating that the low temperature is favorable in the formation of the transition state for [Ch][Sal] in *L*-glycine aqueous solution while the addition of *L*-glycine intensifies the structure breaking behavior of the [Ch][Sal] and releasing of water molecules to the bulk of the solution^42^.

### Electrical conductance results

The ion association and ion solvation behavior and studying the existing interactions and character of ion-pair in a solution could be studies by electrical conductance systematic measurement^43,44^. The specific conductance, *κ* of the solutions containing [Ch][Sal] in water and in aqueous solutions of different concentration of *L*-glycine under atmospheric pressure at different temperatures are given in Table 9.

**Table 9:** The specific conductance (*κ*) of solutions containing [Ch][Sal] in water and in the aqueous solutions of *L*-glycine at different temperatures and *P* = 0.0868 MPa.

10^3^*C*	*κ*	10^3^*C*	*κ*	10^3^*C*	*κ*	10^3^*C*	*κ*
mol L^− 1^	µS cm^− 1^	mol L^− 1^	µS cm^− 1^	mol L^− 1^	µS cm^− 1^	mol L^− 1^	µS cm^− 1^
*T* = 288.15 K		*T* = 298.15 K		*T* = 308.15 K		*T *= 318.15 K	
[Ch][Sal] + water							
0.0682	7.25	0.2034	17.28	0.1623	15.95	0.2667	25.18
0.1512	12.95	0.3906	31.16	0.3853	33.68	0.5138	45.63
0.2193	17.37	0.5533	42.97	0.5354	44.57	0.6275	54.86
0.2771	20.61	0.7160	54.23	0.7545	60.47	0.8510	72.47
0.3423	24.38	0.8869	65.45	0.8802	68.98	1.0863	90.24
0.4119	27.86	1.0253	74.92	1.0465	79.78	1.1726	96.88
0.4786	31.26	1.1758	84.63	1.1926	89.34	1.2158	99.56
0.5467	35.04	1.3548	96.23	1.3751	101.26	1.4001	113.80
0.6133	38.63	1.4931	104.50	1.5495	111.20	1.7060	136.00
0.6815	42.62	1.6599	114.80	1.7523	123.20	2.0158	157.10
[Ch][Sal] + water + *L*-glycine (m_*L*−gly_ = 0.1005 mol kg^− 1^)							
0.0446	25.04	0.1447	21.06	0.1452	15.02	0.1999	28.65
0.0874	27.80	0.3596	36.36	0.3069	27.62	0.4332	47.72
0.1320	30.56	0.6035	53.04	0.5060	42.51	0.6248	62.56
0.1765	33.26	0.7605	63.29	0.6511	52.91	0.8997	83.26
0.2211	35.85	0.9341	74.11	0.7631	60.58	1.1455	101.00
0.2657	38.47	1.1078	84.98	0.9041	70.21	1.4371	122.20
0.3138	41.07	1.3186	97.79	1.0244	78.53	1.6495	136.90
0.3584	43.55	1.5583	111.40	1.2649	94.41	1.8786	152.30
0.4029	45.88	1.7691	122.90	1.3935	102.10	2.0869	166.20
0.4475	48.42	1.9964	135.60	1.5096	109.10	2.2910	179.20
[ Ch][Sal] + water + *L*-glycine (m_*L*−gly_ = 0.2013 mol kg^− 1^)							
0.0329	23.17	0.1809	31.60	0.1698	32.69	0.2052	36.36
0.0658	25.18	0.4153	47.27	0.3728	46.94	0.4513	54.56
0.0987	27.07	0.5798	57.70	0.5302	57.67	0.7344	74.45
0.1332	28.99	0.8060	71.17	0.6752	67.38	1.0175	93.12
0.1710	30.98	0.9951	82.18	0.9113	82.37	1.2719	109.34
0.2122	32.96	1.2007	93.88	1.1391	95.80	1.5345	125.39
0.2516	34.80	1.4022	104.64	1.2634	103.12	1.7848	140.70
0.2862	36.37	1.5832	114.12	1.4498	113.98	2.0433	156.40
0.3273	38.11	1.8258	125.78	1.6403	124.90	2.3018	170.40
0.3651	40.00	2.0396	135.98	1.8102	134.50	2.6095	187.64
[Ch][Sal] + water + *L*-glycine (m_*L*−gly_ = 0.3004 mol kg^− 1^)							
0.0873	29.35	0.2089	39.99	0.1671	39.37	0.3466	56.35
0.1747	34.26	0.4382	54.91	0.3302	50.55	0.5954	73.91
0.2602	39.00	0.6553	68.19	0.4891	61.01	0.9175	95.39
0.3476	43.70	0.8683	80.83	0.6888	73.74	1.2397	115.90
0.4349	48.13	1.1099	94.40	0.8967	86.48	1.3376	121.90
0.5222	52.52	1.3434	107.20	1.0842	97.89	1.5496	135.40
0.6095	56.81	1.5481	118.10	1.2432	107.20	1.8147	151.30
0.6950	60.78	1.7447	128.40	1.4307	118.20	2.0512	165.20
0.7823	64.77	1.9700	140.00	1.5366	124.00	2.3285	180.40
0.8677	68.61	2.1707	150.10	1.6630	131.10	2.6506	198.00

The standard uncertainty for temperature and pressure were *u*(*T*) = 0.1 K and u(P) = 0.001 MPa. The relative standard uncertainties, *u*_*r*_(*κ*) = 0.2, *u*_*r*_(*m*) = 0.002 and *u*_*r*_(*m*_*gly*_) = 0.005 where *m*_*gly*_ is the molal concentration of *L*-glycine in water.

The specific conductance increases with addition of *L*-glycine and the ionic liquid into the solutions. The molar conductivities, *Λ*, of these solutions have been calculated and are given in Table 10.

**Table 10:** The molar conductivities (*Λ*) of the solutions containing [Ch][Sal] in water and in the aqueous solutions with different concentration of *L*-glycine at different temperatures and *P* = 0.0868 MPa.

10^3^*C*	*Λ*	10^3^*C*	*Λ*	10^3^*C*	*Λ*	10^3^*C*	*Λ*
mol L^− 1^	S cm^2^ mol^−1^	mol L^− 1^	S cm^2^ mol^−1^	mol L^− 1^	S cm^2^ mol^−1^	mol L^− 1^	S cm^2^ mol^−1^
*T* = 288.15 K		*T* = 298.15 K		*T *= 308.15 K		*T* = 318.15 K	
[Ch][Sal] + water							
0.0682	74.01	0.2034	77.23	0.1623	83.11	0.2667	89.20
0.1512	71.07	0.3906	75.76	0.3853	81.01	0.5138	86.11
0.2193	69.14	0.5533	74.82	0.5354	78.65	0.6275	85.21
0.2771	66.42	0.7160	73.54	0.7545	76.89	0.8510	83.52
0.3423	64.78	0.8869	72.15	0.8802	75.57	1.0863	81.79
0.4119	62.28	1.0253	71.54	1.0465	73.88	1.1726	81.43
0.4786	60.71	1.1758	70.64	1.1926	72.85	1.2158	80.75
0.5467	60.06	1.3548	69.87	1.3751	71.85	1.4001	80.29
0.6133	59.36	1.4931	68.94	1.5495	70.18	1.7060	78.90
0.6815	58.2	1.6599	68.21	1.7523	68.90	2.0158	77.24
[Ch][Sal] + water + *L*-glycine (m_*L*−gly_ = 0.1005 mol kg^− 1^)							
0.0446	67.97	0.1447	70.86	0.1452	81.06	0.1999	84.67
0.0874	66.26	0.3596	68.57	0.3069	79.42	0.4332	83.10
0.1320	64.79	0.6035	67.11	0.5060	77.63	0.6248	81.37
0.1765	63.73	0.7605	64.99	0.6511	76.40	0.8997	79.59
0.2211	62.6	0.9341	63.70	0.7631	75.39	1.1455	77.98
0.2657	61.96	1.1078	62.54	0.9041	74.10	1.4371	76.88
0.3138	60.74	1.3186	61.22	1.0244	73.53	1.6495	75.89
0.3584	60.1	1.5583	60.22	1.2649	72.11	1.8786	74.90
0.4029	59.22	1.7691	58.60	1.3935	70.98	2.0869	74.06
0.4475	59	1.9964	57.48	1.5096	70.16	2.2910	73.14
[ Ch][Sal] + water + *L*-glycine (m_*L*−gly_ = 0.2013 mol kg^− 1^)							
0.0329		0.1809	70.83	0.1698	73.72	0.2052	76.77
0.0658	65.67	0.4153	68.58	0.3728	71.81	0.4513	75.23
0.0987	63.38	0.5798	67.11	0.5302	70.73	0.7344	73.31
0.1332	61.6	0.8060	64.99	0.6752	69.91	1.0175	71.26
0.1710	59.92	0.9951	63.72	0.9113	68.25	1.2719	69.77
0.2122	58.1	1.2007	62.54	1.1391	66.39	1.5345	68.28
0.2516	56.31	1.4022	61.22	1.2634	65.66	1.7848	67.29
0.2862	54.92	1.5832	60.21	1.4498	64.71	2.0433	66.46
0.3273	53.68	1.8258	58.60	1.6403	63.85	2.3018	65.08
0.3651	52.25	2.0396	57.45	1.8102	63.16	2.6095	64.00
[Ch][Sal] + water + *L*-glycine (m_*L*−gly_ = 0.3004 mol kg^− 1^)							
0.0873	58.85	0.2089	67.12	0.1671	71.27	0.3466	74.55
0.1747	57.53	0.4382	65.65	0.3302	69.94	0.5954	72.90
0.2602	56.83	0.6553	64.17	0.4891	68.59	0.9175	70.71
0.3476	56.08	0.8683	62.99	0.6888	67.19	1.2397	68.88
0.4349	55.01	1.1099	61.50	0.8967	65.82	1.3376	68.33
0.5222	54.22	1.3434	60.34	1.0842	64.96	1.5496	67.69
0.6095	53.49	1.5481	59.40	1.2432	64.14	1.8147	66.56
0.6950	52.62	1.7447	58.61	1.4307	63.43	2.0512	65.66
0.7823	51.85	1.9700	57.80	1.5366	62.83	2.3285	64.37
0.8677	51.17	2.1707	57.11	1.6630	62.32	2.6506	63.19

The standard uncertainty for temperature and pressure were *u*(*T*) = 0.1 K and u(P) = 0.001 MPa. The relative standard uncertainties, *u*_*r*_(*Λ*) = 0.3, *u*_*r*_(*m*) = 0.002 and *u*_*r*_(*m*_*gly*_) = 0.005 where *m*_*gly*_ is the molal concentration of *L*-glycine in water.

**Fig. 4:**
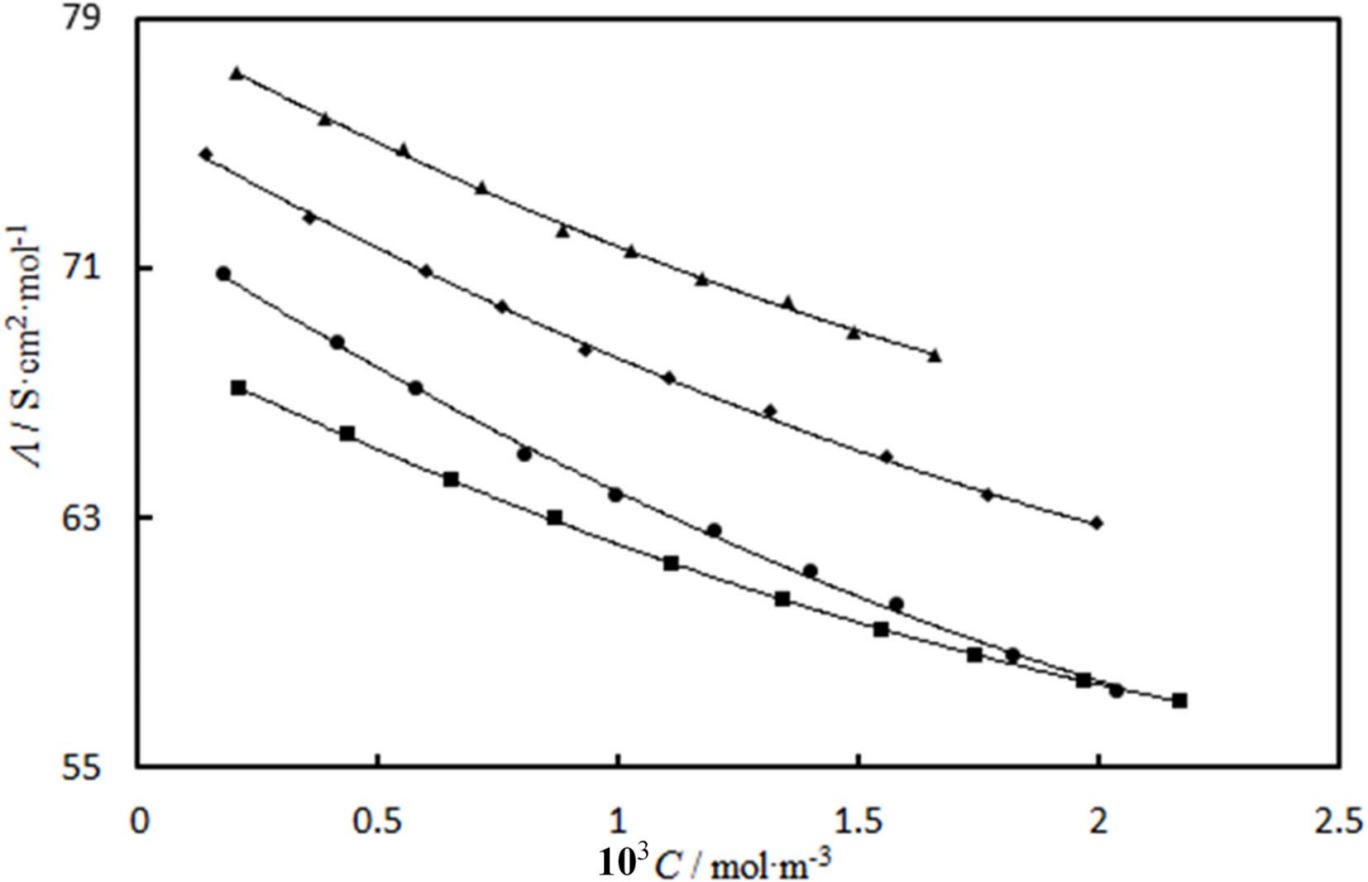
Molar conductivity variation of the aqueous [Ch][Sal] solution with its molarity (*C*) in the presence of different concentration of *L*-glycine: (▲) 0.0000; (♦) 0.1005; (●) 0.2013; (∎) 0.3004 mol kg^− 1^ under atmospheric pressure and at *T* = 298.15 K.

The variation of molar conductivity of these solution with the concentration of [Ch][Sal] in the presence of different concentration of at *T* = 298.15 K are shown in Fig. 4.

The figure show that the values of *Λ* decrease by increasing the concentration of [Ch][Sal] and *L*-glycine at *T* = 298.15 K. An increase in concentration of [Ch][Sal] causes the formation of ion-pairs in the dilute region and stronger ion association in the solutions, also at higher concentration of *L*-glycine the strong attraction between ions of ionic liquid and charge end group of *L*-glycine as zwitterionic compound is created which leads to the lower values of *Λ*. Limiting molar conductivities, *Λ*_*0*_ and ion association constants, *K*_a_, were calculated by correlation of the these data with the low-concentration chemical model (LcCM) uses the set of equations,

16 $$\Lambda =\alpha [{\Lambda _0} - S{(c\alpha )^{1/2}}+Ec\alpha \ln (c\alpha )+{J_1}c\alpha +{J_2}{(c\alpha )^{3/2}}]$$

where ion association constant, *K*_a_ defined as:

17 $${K_a}=\frac{{1 - \alpha }}{{{\alpha ^2}c{\gamma _ \pm }^{2}}}$$

18 $$\ln {\gamma _ \pm }=\frac{{\kappa q}}{{1+\kappa R}}$$

19 $${\kappa ^2}=\frac{{16000{N_A}{z^2}{e^2}\alpha c}}{{{\varepsilon _0}\varepsilon {K_B}T}}$$

20 $$q=\frac{{{z_c}{z_a}{e^2}}}{{8\pi {\varepsilon _0}\varepsilon kT}}$$

where, *Λ* and *Λ*_*0*_ are the molar conductivity and limiting molar conductivity, respectively. (1-α) is the fraction of oppositely charged ions acting as ion pairs, $\ln {\gamma _ \pm }$ is the corresponding mean activity coefficient of the free ions, *κ* is the Debye parameter, *e* is the electronic charge, *z* is the ionic charge, ${\varepsilon _0}$is the permittivity of vacuum, $\varepsilon$ is the dielectric constant of solvent, and the other symbols have been introduced in previous parts. The coefficient *E*, *J*_*1*_ and *J*_*2*,_ are essential for calculations which were taken from reference^45,46^. The parameter *R*, indicate the distance of two centers between ions in the ion pairs. The calculated *Λ*_*0*_, *K*_a_ and *R* for [Ch][Sal] in the aqueous solutions of *L*-glycine are given in Table 11.

**Table 11:** The limiting molar conductivities (*Λ*_*0*_), ion association constants (*K*_*a*_), distance parameters (*R*), and standard deviations (σ (Λ)) of [Ch][Sal] in water and in the aqueous solution of *L*-glycine at different temperature.

T/K	K_a_/(dm^3^ mol^− 1^)	10^4^Λ_0_/(S m^2^ mol^− 1^)	10^8^*R*/(m)	σ (Λ)
[Ch][Sal] + water				
288.15	710.65 ± 0.94	77.65 ± 0.23	0.09 ± 0.01	0.60
298.15	52.55 ± 0.78	79.20 ± 0.25	0.43 ± 0.02	0.14
308.15	60.13 ± 0.86	85.57 ± 0.18	0.50 ± 0.04	0.27
318.15	65.87 ± 0.88	91.33 ± 0.13	0.41 ± 0.01	0.45
[Ch][Sal] + water + *L*-glycine (m_*L*−gly_ = 0.1005 mol kg^− 1^)				
288.15	479.96 ± 0.73	69.02 ± 0.21	0.01 ± 0.00	0.31
298.15	47.93 ± 0.68	76.07 ± 0.26	0.46 ± 0.01	0.12
308.15	53.94 ± 0.34	82.81 ± 0.18	0.47 ± 0.02	0.15
318.15	57.64 ± 0.51	86.48 ± 0.17	0.39 ± 0.01	0.21
[Ch][Sal] + water + *L*-glycine (m_*L*−gly_ = 0.2013 mol kg^− 1^)				
288.15	413.19 ± 0.47	67.85 ± 0.14	0.63 ± 0.01	0.08
298.15	44.03 ± 0.33	72.84 ± 0.17	0.50 ± 0.03	0.15
308.15	50.50 ± 0.28	75.48 ± 0.22	0.47 ± 0.02	0.20
318.15	54.72 ± 0.62	78.95 ± 0.08	0.43 ± 0.05	0.20
[Ch][Sal] + water + *L*-glycine (m_*L*−gly_ = 0.3004 mol kg^− 1^)				
288.15	151.87 ± 0.38	60.08 ± 0.19	0.45 ± 0.01	0.59
298.15	59.62 ± 0.46	68.96 ± 0.23	0.48 ± 0.03	0.11
308.15	64.87 ± 0.61	72.67 ± 0.12	0.43 ± 0.04	0.17
318.15	70.76 ± 0.47	77.38 ± 0.30	0.38 ± 0.02	0.18

The standard uncertainty for temperature and pressure were *u*(*T*) = 0.1 K and u(P) = 0.001 MPa.

It is observed that at higher temperatures *Λ*_*0*_ values are increased which demonstrate the higher mobility of solvated ions as consequence of decreased in viscosity of solution^47^. The values of *Λ*_*0*_ and *K*_a_ decrease at higher concentration of *L*-glycine and this trend are related to an increase in viscosity of solutions and enhancements in the interaction of ions and zwitterionic behavior of *L*-glycine^48^.

### COSMO results and solvation behavior

**Fig. 5:**
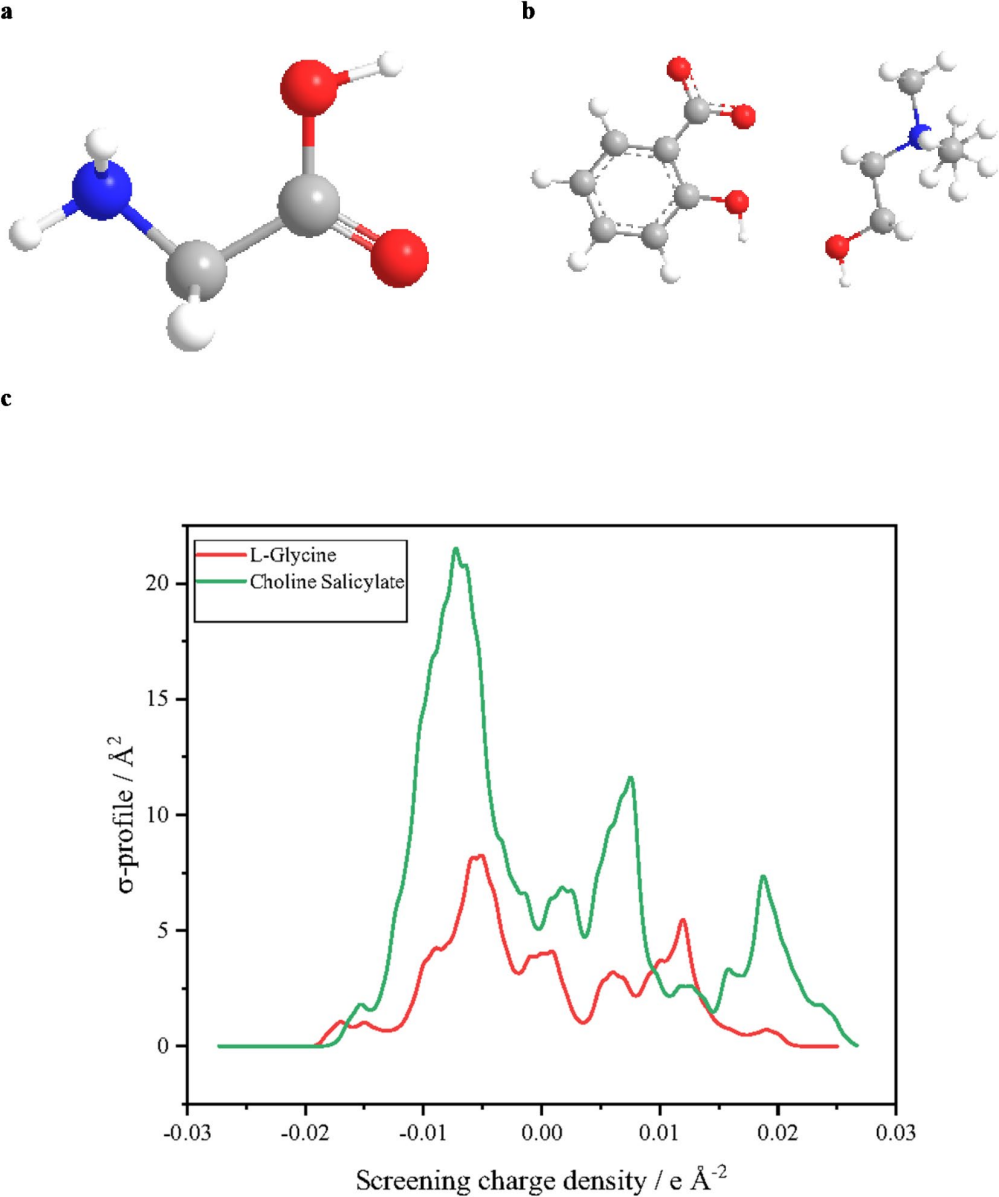
The optimized structure of (**a**) *L*-glycine, (**b**) choline salicylate, and (**c**) Sigma profiles of *L*-glycine and choline salicylate.

In COSMO-based thermodynamics, the *σ*-profile is a unique molecular fingerprint that characterizes charge distribution on the surface. The *σ*-profile is used in COSMO models (e.g., COSMO-RS and COSMO-SAC) to predict thermodynamic properties and interactions. While obtaining *σ*-profiles through density functional theory (DFT) calculations can be computationally expensive, alternative methods and software tools can approximate these profiles for faster analysis. In this study, the GGA VWN-BP function in Dmol^3^ was used to obtain COSMO results, including optimized molecular structures and *σ*-profiles (Fig. 5). Additional results from energy optimization calculations are presented in Table 12.

**Table 12:** The results of COSMO calculations including surface area and total volume of cavity, besides the total HOMO and LUMO orbitals number and energy and dielectric (solvation energy) in water.

Compound	surface area of cavity	total volume of cavity	Dielectric (solvation) energy	*n*_HOMO_	*n*_LUMO_	E_HOMO_	E_LUMO_
	Å^2^	Å^3^	kcal/mol			eV	eV
*L*- glycine	106.97	86.72	−13.06	20	21	−5.70	−1.07
[Ch][Sal]	283.07	286.07	−82.19	65	66	−4.37	−1.15

The comparison of *L*-glycine and choline salicylate structures reveals significant differences. Choline salicylate exhibits a larger molecular size, more complex structure, and greater stability in water compared to *L*-glycine. Electronic properties of choline salicylate demonstrate a less negative HOMO energy, suggesting higher interactions with water compared to *L*-glycine. These findings indicate distinct behaviors in terms of molecular interactions and solubility, providing valuable insights for further analysis and applications. The presence of *L*-glycine might influence the hydration of choline salicylate but is anticipated to have a small impact. However, accurate prediction of hydration behavior in this mixture requires experimental or computational studies to account for factors such as electrostatic interactions, temperature, and concentration.

## Conclusion

In this work, the thermodynamic and transport properties of choline salicylate as the active pharmaceutical ingredient ionic liquid were studied in aqueous solutions containing different concentration of *L*-glycine. The volumetric, compressibility and viscometrical properties revealed strong solute-solvent interactions between [Ch][Sal] in the presence of *L*-glycine, predominantly of the hydrophilic-hydrophilic type interactions. The electrical conductometric analysis showed decreased ion mobility and limiting molar conductivities (*Λ*_*₀*_) for aqueous solutions of [Ch][Sal] in the presence of higher concentrations of *L*-glycine, attributed to increased solute-solvent interactions. The Hepler’s constant confirmed the structure-breaking properties of [Ch][Sal] in the presence of *L*-glycine. The COSMO results suggests that *L*-glycine has a limited impact on the hydration of [Ch][Sal]. These findings provide valuable insights into the behavior of [Ch][Sal] in the aqueous solutions in the presence of *L-*glycine as a simple simulated biological media for active pharmaceutical ingredient ionic liquid behavior.

## Electronic supplementary material

Below is the link to the electronic supplementary material.

Supplementary Material 1

## Data Availability

All data generated or analyzed during this study are included in this published article [and its supplementary information files].
