# Supplementary material for: Theoretical and experimental investigation of hydration behavior of choline salicylate ionic liquid in the presence of L- glycine
Source: Sci Rep. 2025 Apr 22;15:13867. doi: 10.1038/s41598-025-95345-8 (PMC12015446; doi:10.1038/s41598-025-95345-8)
Supplement: Supplementary file 1 — Supplementary Material 1 [file 41598_2025_95345_MOESM1_ESM.docx]

**Supporting information**

**Theoretical and experimental investigation of hydration behavior of choline salicylate ionic liquid in the presence of *L*- glycine**

Negin Ammari, Behrang Golmohammadi, Fariba Ghaffari, Hemayat Shekaari^*^, Mohammed Taghi Zafarani-Moattar,

Department of Physical Chemistry, University of Tabriz, Tabriz, Iran

^*^Corresponding author. Tel.: +984133393094. Fax: +984133340191.

E-mail address: hemayatt@yahoo.com (H. Shekaari).

**Characterization of Choline Salicylate [Chol][Sal]**

The synthesized API-IL was characterized by ^1^H, ^13^C NMR and IR. The proton NMR of [Chol][Sal] was recorded on Brukar Avance 400 MHz spectrometer H NMR 400 MHz. ^1^H NMR (DMSO) : 7.694 (d, 1H, CH-CH=C-COO-), 7.153(t, 1H, CH-CH=C-COO-), 6.596-6.620 (m, 2H, CH-CH=C-OH), 5.174 (s, 1H, CH_2_OH), 3.863 ( t, 2N, N-CH_2_ ), 3.442 (t, 2H, CH_2_-O-CO-), 3.136(s, 9H, N-(CH_3_)_3_, 2.494(s, 1H, CH2OH). IR (KBr): ν 684.63, 953.19 cm^–1^ (stretching vibration peak approves the position of the ortho in the aromatic ring), 1143.77 cm^-1^ (bending vibration peak of C-N), 1584.98 cm^–1^ (bending vibration peak of N–H), 1754.19 cm^–1^ (stretching vibration peak of C=O ), 1754.19-1948.60 cm^–1^ (skeletal vibration peaks of CO-OH), 3298.00 cm^–1^ (stretching vibration peak of aromatic ring), 2500-3000cm^–1^ (stretching vibration peak of C-H).


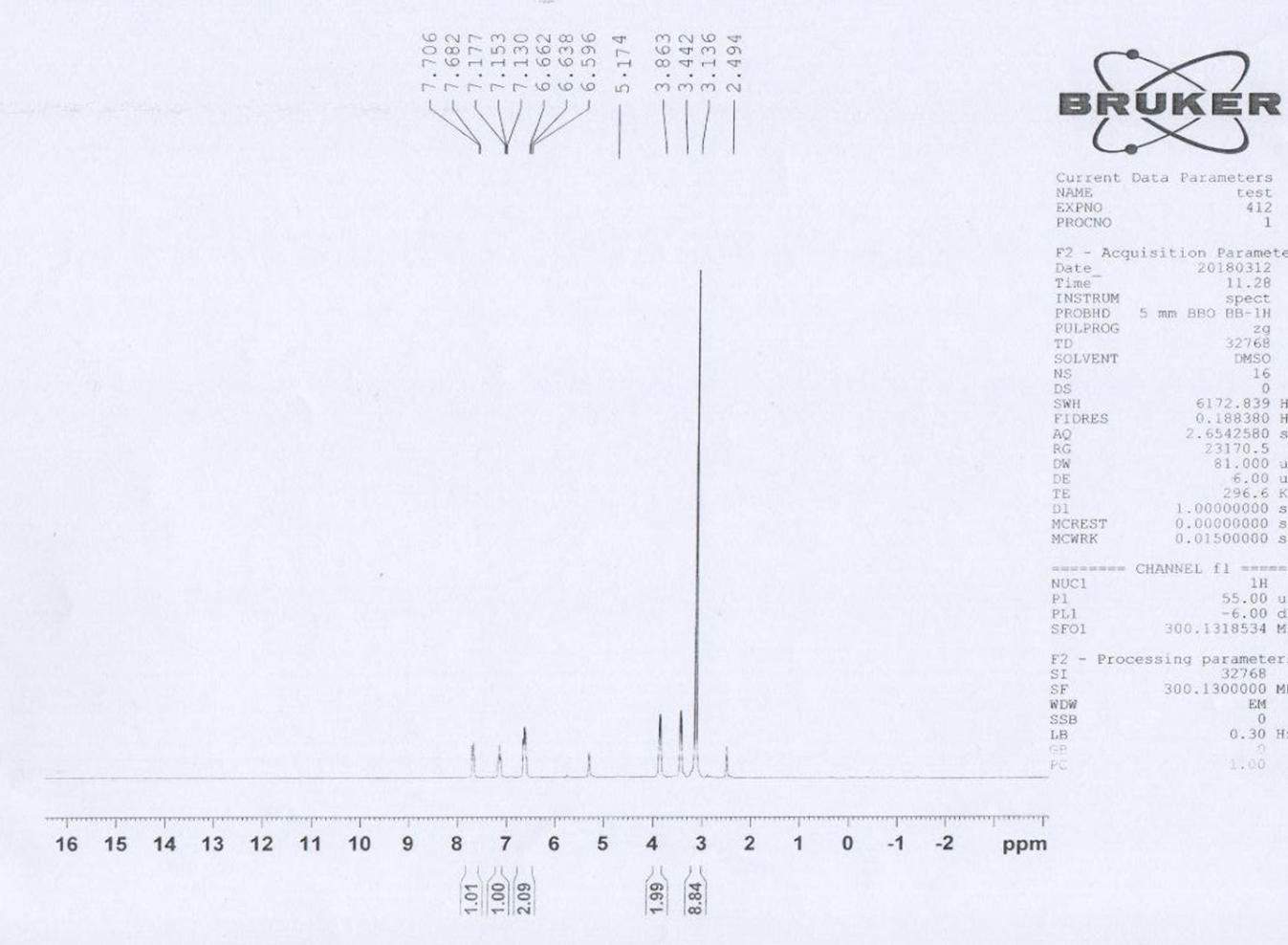


**Figure S1.** 1H NMR spectrum (DMSO, 400 MHz) of [Chol][Sal]


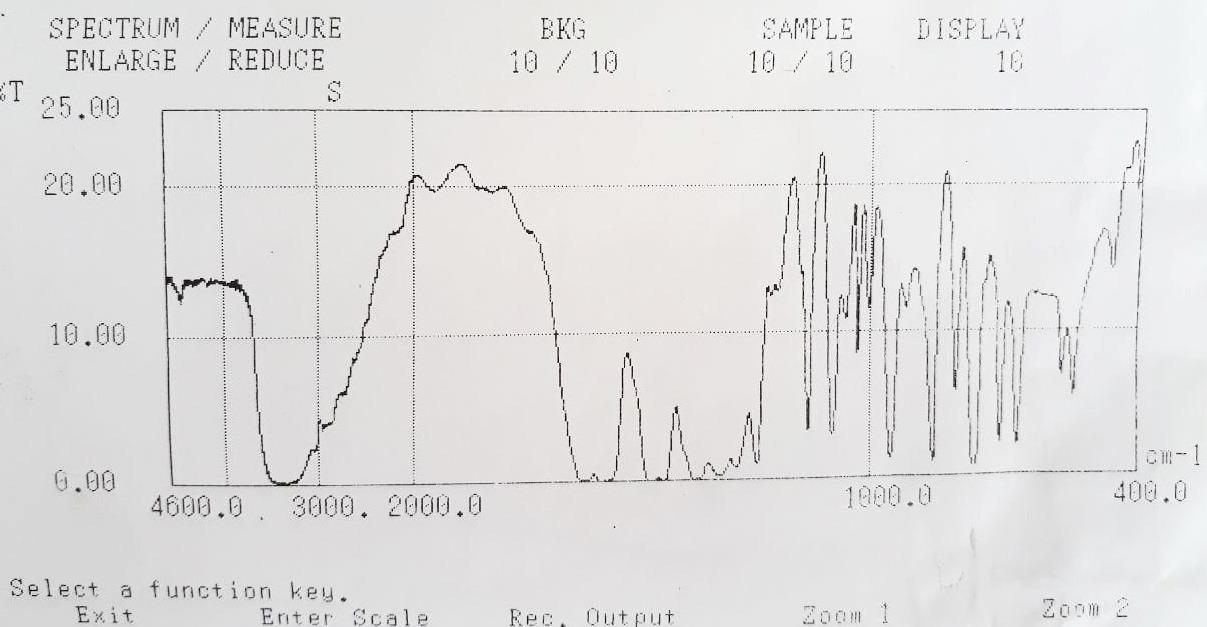


**FIGURE S2.** IR spectrum (KBr) of [Chol][Sal]
